# Intrinsic motivation in a virtual reality mock crime affects participants’ willingness to invest more effort in deceptive interviews

**DOI:** 10.1038/s41598-023-50515-4

**Published:** 2024-01-02

**Authors:** Isabella S. Branson, Craig P. Speelman, Shane L. Rogers

**Affiliations:** Experimental Psychology Unit, Edith Cowan University, Perth, WA Australia

**Keywords:** Health care, Signs and symptoms

## Abstract

In studies of investigative interviewing, it is not well understood how participant experience of mock-crime activities might affect participants’ desire to perform (well) in subsequent interviews. In this study, we utilized two immersive virtual reality mock-crimes to examine if participants’ intrinsic motivation (i.e., competence, autonomy, relatedness) while committing the virtual mock-crime affects their desire to perform well in interviews. We also examined if the self-reported feeling of presence during the virtual reality mock-crime is associated with participants’ intrinsic motivation. We found significant positive associations between presence and all intrinsic motivation variables in both truth and lie conditions. We also found that competence and relatedness significantly predicted the self-reported effort to perform well in interviews. We discuss these results in the context of prior literature and provide recommendations for researchers on the design of mock-crime experiences.

## Introduction

An investigative interview is a process where individuals are questioned using various known or novel interview techniques (e.g.^1–3^) for the purposes of gathering or eliciting information from interviewees within an intelligence, forensic, or criminal context (e.g.^1,4–10^). As such, there are several types of interviewees including real-world detainees, witnesses, victims, and suspects, or students/general population playing these roles. In research studies, investigative interviews can be conducted in the field (i.e., real-world settings) or within laboratory environments^11^. Mentions of investigative interviews in current study will refer specifically to investigative interviews that occur in laboratories examining deception by students/general population playing the role of a suspect after a mock crime.

In laboratory experiments involving investigative interviews, participants follow a procedure where they are first instructed to commit a mock crime prior to being interviewed by a researcher (e.g.^12,13^). Due to ethical considerations, participants typically experience mock-crime activities that are non-violent such as a theft (e.g.^4,14^) or burglary (e.g.^8,9, 15^). Mock crimes are designed to help prepare participants for an interview by providing participants with real memories/knowledge of the simulated crime activity whilst immersing them into a mock-suspect role. During the post mock-crime interviews, participants may be asked a variety of questions pertaining to the events of the mock-crime, however, they are most commonly instructed to either tell the truth about the performed activity or create a convincing narrative that conceals details of the crime and/or their involvement.

To encourage participants to perform well in investigative interviews, researchers extrinsically motivate participants to successfully deceive the interviewer with monetary incentives (e.g.^13,16^) or intrinsically motivate participants by issuing a challenge to successfully deceive an interviewer or a lie detector test (e.g.^14,17, 18^). While many researchers take steps to actively encourage participants to engage with the interview component^19^, examining participant motivation whilst engaging with the initial mock-crime component is generally overlooked. In this study, we examine if the intrinsic motivation experienced by participants within the mock-crime differ between truth/lie conditions. We also examine if participants’ intrinsic motivations affect how participants intend to perform in subsequent interviews.

Within offender literature, the motivation to commit a burglary may simultaneously stem from both an extrinsic and intrinsic motivator. For many offenders, financial gain was identified as the extrinsic motivator for committing a burglary^20–22^. However, in interviews with burglars about their modus operandi, factors akin to intrinsic motivation can be found in the initiation and continual perpetration of the crime (e.g., boredom relief, enjoyment derived from the thrill of the crime, satisfaction after each heist)^11,12^. Additionally, factors that enhanced their intrinsic motivation can also be found whilst committing the crime (e.g., social acceptance/interaction, ability to selectively pick target properties and items, acquiring expertise)^8,23^. Although the presence of intrinsic motivation, and its individual predictors of competence, autonomy, and relatedness are reflected within the offender population, it has not been considered within mock-crime research.

Given that a core function of a mock-crime experience is to act as a simulation of a real crime, it may be worthwhile for researchers to examine how participants’ intrinsic motivation in a mock-crime can affect how the participant performs in later activities. Specifically, we argue that participant intrinsic motivation to engage with the mock-crime could potentially influence how participants behave in subsequent investigative interviews. We believe that this line of inquiry may be of interest to researchers using mock-crimes and may help inform future experimental designs of research involving mock-crimes. To examine this assertion participants in the present study self-reported their level of intrinsic motivation for the mock-crime component. We then examined how this influenced the amount of effort participants were willing to invest in performing well in the subsequent interview phase.

Prior deception detection research has suggested that being deceptive is associated with providing less narrative and reminiscent details^24^, less externally verifiable details^25^, less internally (i.e., contextual, perceptual) verifiable details^12^, exhibiting different thermal facial changes^26^, slower response times^27^, higher cognitive load^28–32^, and displaying different frequencies of facial and body expressions^33^ compared to truthtellers. Considering this research suggesting differences in truth/lie tellers, in the present study we decided to additionally investigate participants’ self-reported level of intrinsic motivation for the mock-crime component across conditions that differed on instructions to be truthful or deceptive.

### Participant motivation for performing mock-crimes in research studies

Self-Determination Theory (SDT)^34,35^ is a popular psychological theory of motivation that makes a distinction between external and intrinsic motivation. Extrinsic motivation refers to engagement with an activity that is motivated by a separate instrumental outcome (i.e., external source), where the instrumental outcome is dependent on the level of autonomy held by the individual^36^. For example, a person might be motivated to put effort into their studies primarily to satisfy their parents (i.e., an external motivator). Whereas intrinsic motivation refers to an internally regulated engagement of the self with an activity, which drives attempts to produce satisfaction in/for the self^34^. This can manifest as curiosity, mastery, or exploration towards a positive experience with the task^36^. For example, a person might be motivated to put effort into their studies primarily to satisfy their own personal interest in the subject matter (i.e., an internal motivator).

The notion of intrinsic motivation has been postulated to contain three core needs (i.e., competence, autonomy, relatedness) that contribute to the enjoyment or interest in an activity^35^. Competence refers to the ability to successfully perform a behaviour to elicit a desired outcome, autonomy refers to the ability to initiate or claim ownership over the performance of a behaviour, and relatedness refers to interpersonal connections formed while performing a behaviour/activity^35,37^.

According to the SDT, the amount of effort that an individual is willing to dedicate to a task is dependent on the fulfilment of perceived competence, autonomy, and relatedness^38–42^. For example, higher intrinsic motivation in students has been found to positively associate with academic performance^43^. Also, employees with higher levels of intrinsic motivation were typically found to have higher levels of engagement with their work^44^. Despite the relatively widespread acknowledgement of the importance of intrinsic motivation for task engagement, the notion of intrinsic motivation for participation in mock crimes has been largely absent in research investigating criminal interviewing.

### Increasing intrinsic motivation to perform mock-crimes using virtual reality

The mock-crimes used in research examining investigative interviewing techniques typically require participants to physically perform specific actions at a location on a university campus. If the mock-crime or interview/examination room do not mimic real-world aspects of a crime, this may have a negative impact on participant immersion, presence, and motivation with the experience^45^. Feelings of perceived immersion and presence have long been recognised as important factors that facilitate engagement and performance with a role-playing activity^46,47^.

In recent years researchers have turned to virtual reality to create mock-crime scenarios that are otherwise not possible to enact (e.g.^15,23, 45, 48–52^). By creating a gamified type of experience participants are expected to be more engaged when role-playing their part, which should facilitate their immersion and intrinsic motivation to perform the task^53–56^. In the context of virtual reality, a sense of immersion can be defined as how well the hardware (e.g., head-mounted device) mimics how we experience reality through sensory inputs^57^. Whereas a sense of presence can be defined as how much an individual feels like they are a part of the virtual environment^58^.

A recent study^45^ examined the utility of virtual reality for the presentation of a mock-crime. Participants in the study were assigned into two different conditions (innocent or guilty) and two different modes of mock-crime presentation (real-world or virtual reality). Innocent participants entered an office and gift-wrapped a present to be given to their professor. Guilty participants had to turn off a closed-circuit television camera and gift-wrap the present while discreetly stealing the item inside. The researchers built a virtual reality replica of the real-world mock-crime office. Participants in the virtual mock-crime condition rated the virtual environment at an average of 9.1 out of 10 on a perceived realism measure.

An earlier study^50^, compared the behaviours of six experienced ex-burglars, and six non-offender students as they navigated and stole items from a real house and a 3D simulation of the same house (presented on a laptop). In the real house, participants were asked to burgle the property and indicate which objects they would have taken. Participants were then asked to complete the same task on the 3D simulation of the house. The researchers found that each group of participants behaved similarly in both the real and simulated house. This led to a subsequent study where the authors^49^ created a small virtual neighbourhood (presented on a laptop) to compare burglarising expertise between experienced convicted burglars, convicted offenders of other crimes, and non-offenders. Participants were asked to explore the neighbourhood and choose a property to burgle. In all three groups, participants indicated high levels of presence (a feeling of being in the virtual environment) while performing the task.

### The current study

Prior studies making use of virtual mock-crimes have indicated that the use of virtual reality mock-crimes can be immersive and engaging for participants^15,23, 45, 48–52^. However, a current gap in the literature is understanding how participants’ levels of intrinsic motivation and presence in the mock crime experience might impact upon their subsequent effort to perform in the interview component. In the current study participants performed two mock-crimes in virtual reality. After each mock-crime experience they were asked to be either truthful or deceptive in a subsequent interview. Self-report measures were obtained regarding participants’ perceived intrinsic motivation for performing the mock-crimes, their sense of presence with the mock-crimes, and their self-reported intent to put in effort (hereby referred to as intended effort) to perform in the subsequent interview phase of the procedure.

One aim of the current study is to explore associations between presence and intrinsic motivation in the context of mock-crime experiences in virtual reality. Prior research on educational applications with virtual reality has reported positive associations between immersion, presence, and intrinsic motivation^54,59, 60^. As such, we hypothesise that there would be positive relationships between the aspects of intrinsic motivation (i.e., competence, autonomy, and relatedness) and presence.

Another aim of the current study is to better understand how intrinsic motivation while performing a mock-crime relates to intended effort to perform in a subsequent interview component. Considering the generally accepted notion that higher levels of intrinsic motivation are associated with greater levels of effort to perform tasks^38,39^, we expected this would also be the case in the specific context of mock-crime experiences. As such, we hypothesise that higher reported levels of intrinsic motivation while performing the mock-crime would be associated with higher reported levels of intended effort to perform well in the interview phase.

In this study we separate intrinsic motivation into the core components of competence, autonomy, and relatedness to explore which facet of intrinsic motivation might be the strongest predictor of intended effort to perform in the interview. Following a commonly used paradigm, we examine this separately for an interview where the participant is required to be truthful versus an interview where the participant is required to be deceptive. Within the context of an investigative interview, the distinction between truth-telling and lying participants is particularly important as deceptive individuals (compared to truth-telling individuals) are thought to not only produce different verbal, linguistic, and non-verbal behaviours, they may also be under a higher cognitive load when lying and adopt different interrogation strategies to appear honest (e.g.^19,61–64^). We hypothesise that participants in the lie condition would report significantly higher levels of intended effort compared to when they were telling the truth.

## Methods

### Participants

Initial data collection included 101 participants. However, two participants were excluded from the sample for not following instructions. The final sample was 99 (*M*_age_ = 28.41, *SD* = 10.40) university psychology students (65 females, 33 males, and 1 non-binary). Participants were recruited through advertisements on SONA, a website that manages the recruitment of undergraduate students, and reimbursed with 2 credit points as part of the undergraduate research participation scheme within the psychology degree. All participants were recruited in compliance with national ethical and professional standards for human research as outlined in the National Statement on Ethical Conduct in Human Research (2007), Australia. Informed consent was obtained from all participants. Research ethics approval was obtained via the Human Research Ethics Committee of Edith Cowan University (Low risk—Approval number: 2020-01677-BRANSON).

### Interviewer

Only one interviewer was used for all interviews. The interviewer had no formal training and read the interview questions from a prepared script. A full transcript of the standard interview questions can be found at: 10.6084/m9.figshare.16807444.v8^65^.

### Equipment

The virtual reality simulations in this study were designed and run via the software Unreal Engine 4 (UE4) on a desktop computer with specifications: PU (Intel i7-9700K), GPU (Nvidia RTX 2080), RAM (32 GB DDR4), Storage (500 GB SSD and 2 TB HDD). The VR head mounted display (HMD) unit used was an Oculus Rift S. This is a tethered headset with 1280 × 1440 resolution per eye with an 80 Hz refresh rate that connects to the PC via the DisplayPort.

### Procedures

**Figure 1:**
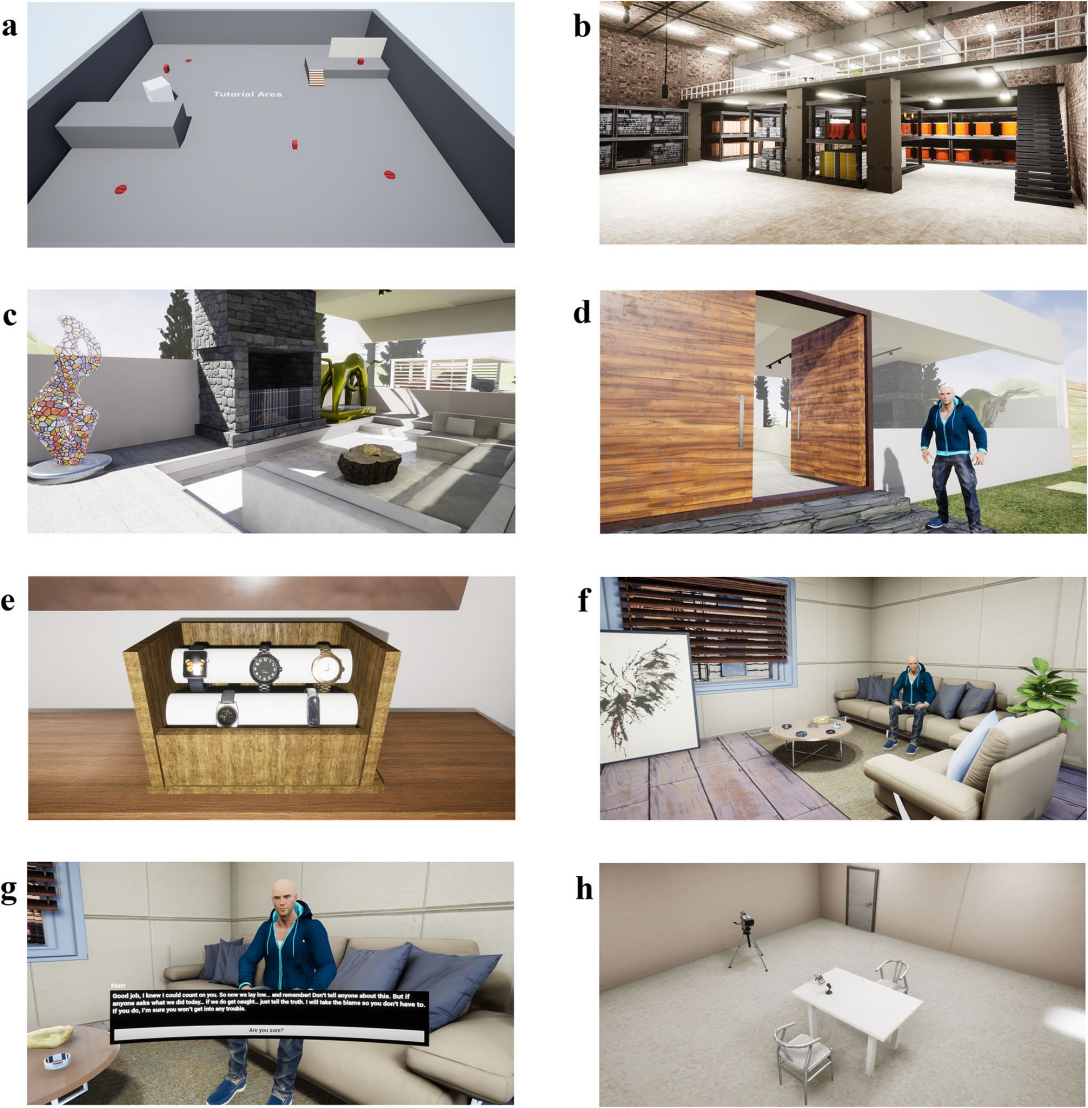
This presents examples of what the participant experiences when in virtual reality. (**a**) Is the tutorial area. It is the first level that participants experience. In this level, participants learn how to move around and grab objects in virtual reality. (**b**) Shows the warehouse environment. The bottom floor is the warehouse, and the top floor is an office. (**c**) Shows the living room area of the house environment. (**d**) Shows Matt, the in-simulation confederate standing outside the house environment. (**e**) Is an example of an item that can be stolen from the house environment. (**f**) Shows the environment of Matt’s apartment. (**g**) Shows Matt seated in front of the participant in his apartment. Here, Matt gives the participant instructions on what to do for the interview. (**h**) Shows a bird’s eye view of the interview room that participants would be sitting in while they concoct a lie, or rehearse their truth account.

Upon providing informed consent, participants were seated and asked to put on the Oculus Rift HMD. Participants were then guided through an initial tutorial stage to get the participant acquainted with moving around within the virtual environment (see Fig. 1a). This section of the experiment took approximately 5 to 10 min to complete. A within-subjects design was used where all 99 participants engaged in a mock-crime scenario in VR with a subsequent interview. This was repeated with a second VR mock-crime scenario and a final interview. Participants were randomly allocated to experience the truth condition (i.e., warehouse scenario), or the lie condition (i.e., house scenario) first.

### Procedure: virtual reality mock-crime simulation

Participants experienced two similar virtual mock-crime scenarios that took place in different locations. These locations were a warehouse (Fig. 1b) and a house (Fig. 1c). Each scenario was counter-balanced across participants. For both scenarios the participants were told that they were playing the role of Matt’s best friend. Matt is the in-simulation confederate represented by an avatar as a non-player character (NPC) (see Fig. 1d). Matt had idle animations (i.e., blinks, breathes, or sways slightly on the spot) to indicate that he is not dormant. In each scenario, Matt was positioned at the entry/exit point of the venue. Matt instructed the participant to steal three items from the venue while he stood watch. After acknowledging Matt’s instructions, the participant was able to roam the venue in search of the items (see Fig. 1e). This took approximately 5 to 10 min to complete.

After the participant found all the items, they returned outside to Matt who informs the participant that they are leaving. The scenario transitioned to Matt’s living room in his apartment where the participant was seated across from Matt (Fig. 1f). A short-structured conversation with Matt ensued, where Matt informed the participant how to act should they be brought in for questioning (Fig. 1g).

In the truth condition Matt relieves the participant of all responsibility and encourages them to tell the truth. In the lie condition Matt encourages the participant to tell a convincing narrative that would absolve any suspicions towards the pair (i.e., instructs the participant to be deceptive). After the participant indicated they had understood the instructions a text box informed them that the police had requested an interview. This section of the experiment took approximately 3 min to complete.

The scenario subsequently transitioned to an interview room where participants were provided five minutes to either think about what they did (i.e., truth condition) or concoct a fictional narrative (i.e., lie condition) (Fig. 1h). Participants were not informed about the nature of the questions that would be asked. Participants were given an on-screen prompt that included a text-box that reminded participants of their instructions (i.e., to be deceptive or truthful in the interview), and a countdown timer starting from 5:00 min. Once the timer reached zero, participants were notified to remove the HMD to begin the interview.

### Procedure: interview

Upon removing the HMD, the participant was interviewed by the experimenter (IB) face-to face. The experimenter role-played being an investigator for the local police force.

Participants were asked questions across different interview phases based on the Cognitive Interview for Suspects^66^. These phases were: Rapport building, first narrative, second (reverse) narrative, and third narrative. In the initial rapport building phase participants were asked questions such as “How has your day been?”. In the first narrative phase participants were informed that Matt had been sighted at the location of the crime. The experimenter also informed participants that they are aware that the participant and Matt are acquaintances, and that the participant had been seen by Matt’s neighbours at his house the same day. The experimenter then asked the participant to provide a detailed account of their day, centred around their meeting with Matt.

In the second (reverse) narrative phase the participant was asked to retell their account a second time in reverse-order. In the third narrative phase the participant retold their account in chronological order once more. This section of the experiment took approximately 10 min to complete. A full transcript of the standard interview questions can be found at: 10.6084/m9.figshare.16807444.v8^65^. The interviewer followed the same script across all the participants and endeavoured to maintain a consistent demeanour while interacting with all participants.

### Procedure: post-interview

After each interview participants completed a survey that included two self-report basic demographic questions about familiarity and experience with virtual reality, a presence questionnaire, and an intrinsic motivation questionnaire. For the two questions about participants’ familiarity and experience with virtual reality, participants were asked “Do you have any personal experience with virtual reality? (e.g., playing games in virtual reality)” and “How would you rate your familiarity with virtual reality?”. Participants answered on a 5-point Likert scale (where 1 = Not at all to 5 = A great deal).

The Presence Questionnaire^67^ consisted of 23 items from the original 29 item questionnaire measuring involvement, visual and haptic fidelity, audio fidelity, immersion, interface quality, and consistent with expectations (virtual environment reactions to participant action). According to the authors, presence is an amalgamation of, and is also affected by these variables. In this study, six questions measuring the sensory fidelity of sound and touch were removed for this study as neither sense were used in the current virtual reality mock-crime. Items were rated on a 7-point scale (where 1 = Not at all to 7 = Very much). Scores could range from 23 to 161. Higher total scores indicate greater levels of presence from participants towards the virtual reality scenario.

Participants completed a shortened version of the Post Experimental Intrinsic Motivation Inventory (PEIMI)^68^ which consists of seven subscales. Participants answered on a 7-point Likert scale (where 1 = Not at all to 7 = Very much). The shortened version used four (i.e., Perceived competence, Perceived autonomy, Relatedness, and Effort) of the seven subscales, and consisted of three positively worded questions in each subscale. The wording of each question was adapted to better fit the needs of the research. Examples of questions from each subscale are as followed, perceived competence (“I think I was good at committing the virtual reality mock-crime), perceived autonomy (“I believe that I had some freedom to perform activities/interactions within the virtual reality simulation”), relatedness (“I felt like I was valued by Matt”), and intended effort (“Prior to the interview, I had intended to try very hard to do well at ‘telling the truth’ *or* ‘lying’ in the interview”). A composite subscale score was created by summing the three individual questions. For each subscale, scores ranged from 3 to 21. Full copies of all questionnaires used in this study can be found at: 10.6084/m9.figshare.16807444.v8^65^.

## Results

Our main purpose in this study was to examine how different aspects of intrinsic motivation predicted intended effort to perform in subsequent interviews. We hypothesised that higher reported levels of competence, autonomy, and relatedness would be associated with higher reported levels of intended effort to perform well in the interview phase. We also hypothesised that higher reported levels of competence, autonomy, and relatedness would be positive associated with higher reported levels of presence. We were additionally interested in how intended effort and the variables of intrinsic motivation were affected by the interview condition (i.e., truth or lie), and hypothesised that participants in the lie condition would report significantly higher levels of intended effort compared to when they were telling the truth. Participants only reported having little experience (M = 1.86, SD = 0.84) and slight familiarity (M = 2.08, SD = 0.94) with virtual reality. Descriptive statistics for competence, autonomy, relatedness, intended effort, and presence are presented in Table 1. The raw data for this article are available at 10.6084/m9.figshare.16807444.v8^65^.

**Table 1:** Descriptive statistics for measures of intrinsic motivation, intended effort, and presence.

	M	SD	Skew	Kurtosis	M	SD	Skew	Kurtosis
	Truth condition				Lie condition			
Competence	15.83	4.12	− 0.46	− 0.70	15.48	4.44	− 0.75	0.05
Autonomy	12.34	4.42	− 0.01	− 0.84	12.70	4.33	− 0.46	− 0.44
Relatedness	13.03	5.41	− 0.28	− 1.02	9.41	4.92	0.36	− 1.01
Effort	15.13	5.20	− 0.82	− 0.14	15.68	4.26	− 0.69	0.23
Presence	109.25	15.11	− 0.08	0.06	110.80	15.11	− 0.52	− 0.07

### Comparisons between intrinsic motivation and intended effort across truth and lie conditions

The mock crime experiences across truth and lie conditions were very similar, by design. In both conditions the participant was required to move around a location in VR and steal several items. The only meaningful difference between conditions was at the end of the experience where the in-simulation partner in crime (i.e., Matt) instructed the participant to either be truthful (truth condition) or deceptive (lie condition). Due to the similarity between conditions, we did not expect differences for presence or intrinsic motivation across conditions.

Participants reported a high level (highest possible score = 161) of presence for both truth (*M* = 109.25, *SD* = 15.11) and lie (*M* = 110.80, *SD* = 15.11) conditions with no significant differences in presence between the truth and lie virtual reality mock-crimes, *t*(98) =  − 1.46, *p* = 0.15, *d* =  − 0.15. There were also no significant differences for perceived competence, *t*(98) = 0.85, *p* = 0.40, *d* = 0.09, or autonomy, *t*(98) =  − 1.08, *p* = 0.28, *d* =  − 0.11. There was however a significant difference for relatedness where a higher mean was found for the truth condition, *t*(98) = 6.76, *p* < 0.001, *d* = 0.68. This last finding was unexpected, and we provide some possible explanations for this in the “Discussion” section.

As lying is generally accepted to be more cognitively demanding than telling the truth, we expected a significantly higher level of intended effort to perform in the interview component for the lie condition. However, we did not find any significant difference between the truth and lie conditions for intended effort to perform in the interview stage, *t*(98) =  − 0.90, *p* = 0.37, *d* = 0.09. We provide possible explanations for this in the “Discussion” section.

### Relationship between measures of intrinsic motivation and presence

The relationship between self-reported presence and intrinsic motivation for mock crimes has not been examined previously. Therefore, this was an exploratory aspect of our study. As revealed in Table 2, there are significant moderate positive associations between intrinsic motivation variables and immersion/presence.

**Table 2:** Spearman correlations between presence, intended effort, and variables of intrinsic motivation.

	Presence	Effort	Competence	Autonomy
Truth condition				
Presence				
Effort	0.04			
Competence	0.52**	0.08		
Autonomy	0.50**	0.18	0.34**	
Relatedness	0.32**	0.22*	0.14	0.28**
Lie condition				
Presence				
Effort	0.27*			
Competence	0.51**	0.25*		
Autonomy	0.49**	0.23*	0.34**	
Relatedness	0.52**	0.31*	0.38**	0.31*

*p < 0.05, **p < 0.001.

### Relationships between intrinsic motivation for the mock crime and intended effort to perform in the interview stage

A major aim of the current study was to better understand how intrinsic motivation to perform the mock crime might be associated with intended effort to perform in the interview component. As can be seen in Table 2, only relatedness was positively associated with intended effort in the truth condition, whereas competence, autonomy, and relatedness were positively associated with intended effort in the lie condition. To further examine how feelings of competence, autonomy, and relatedness predicted how much effort participants intended to exert in the interview for the lie condition, a multiple regression analysis was performed. The overall regression was statistically significant (R^2^ = 0.15, *F*(3, 95) = 5.66, *p* < 0.05. Only competence (*β* = 0.22, *p* < 0.05) significantly predicted intended effort when controlling for other intrinsic motivation variables, whereas autonomy (*β* = 0.09, *p* > 0.05) and relatedness (*β* = 0.19, *p* > 0.05) were non-significant. A post hoc power analysis was conducted using G*Power^69,70^. An *f*^2^ of 0.18, α = 0.05, sample size of 99 and 3 predictor variables were entered. The results of the post hoc power analysis show a 1 − β error probability of 0.95, suggesting adequate power.

As the variables in this study were consistently negatively skewed, we re-ran the regression analysis after performing a squared (*x*^2^) transform on *all* variables. The overall regression for participants in the lie condition using transformed variables was statistically significant (*R*^2^ = 0.14, *F*(3, 95) = 5.16, *p* < 0.05). However, this time only relatedness (*β* = 0.22, *p* < 0.05) was found to be a significant predictor (after controlling for other intrinsic motivation variables), whereas competence (*β* = 0.16, *p* > 0.05) and autonomy (*β* = 0.11, *p* > 0.05) were not statistically significant. A post hoc power analysis was conducted using G*Power (Faul et al. 2007, 2009). An *f*^2^ of 0.16, α = 0.05, sample size of 99 and 3 predictor variables were entered. The results of the post hoc power analysis show a 1 − β error probability of 0.92, suggesting adequate power.

Taking both analyses into account, we note that the standardised beta values for both competence and relatedness were very similar across both analyses (i.e., around 0.20). Overall, the results suggest that of the three intrinsic motivation variables, feelings of competence and relatedness in the mock-crime appear to be the most important predictors of intended effort to perform in the interview phase when a participant is asked to be deceptive.

## Discussion

Prior mock-crime studies typically focus on providing motivation for participants to engage with a subsequent interview component^13,16–19^. However, participants’ intrinsic motivation experienced *while performing a mock-crime* has received little research attention. We hypothesised that higher levels of intrinsic motivation while performing a mock-crime in virtual reality would be positively associated with how much intended effort participants are inclined to invest in a subsequent interview.

### Relationships between intrinsic motivation and intended effort

Our hypothesis that higher reported levels of competence, autonomy, and relatedness would be associated with higher reported levels of intended effort to perform well in the interview phase was partially supported. Partial support was found because in the lie condition all three components of intrinsic motivation (i.e., competence, autonomy, and relatedness) were positively associated with intended effort. A follow-up regression revealed that among the variables of intrinsic motivation, competence and relatedness appeared to be equally the most influential in promoting intended effort to do well in deceptive interviews. However, in the truth condition, only relatedness was associated with intended effort. Therefore, as expected, fostering intrinsic motivation to perform mock-crimes may have an influence on intended effort in the interview phase.

Relatedness to the in-simulation confederate (i.e., Matt) was found to be a significant predictor of intended effort in the subsequent interview. Overall, there was a significantly higher level of relatedness felt towards Matt in the truth condition compared to the lie condition. The interaction between Matt and the participant was scripted and identical in both conditions until Matt gives instructions to the participant pertaining to the upcoming interview. Post-procedure discussions with participants provided us with some anecdotal evidence that some participants felt forced by Matt to lie when that would not necessarily be their natural reaction. Such emotions may have been exacerbated because Matt was portrayed as a close friend. Researchers who are interested in having an in-simulation confederate in VR mock crimes may wish to consider trying to counteract any negative feelings towards the in-simulation confederate associated with being placed into a position requiring them to be deceptive.

Prior research has found that having more interaction with non-player characters can increase a sense of relatedness via fostering parasocial bonds. For example, a qualitative study^71^ found that players in the video game ‘Animal Crossing’ developed relatedness satisfaction via interactions with non-player characters. These parasocial relationships also positively affected their general sense of belonging (or presence) within the virtual world. Another study^72^ found that relatedness was the strongest predictor (i.e., out of competence, autonomy, relatedness, intuitive controls, and presence/immersion) for the number of hours invested in a video game, regardless of the genre of the video game. Their study included a large portion of games that only featured non-player characters.

Therefore, in virtual mock-crime experiences, one possible solution to foster more relatedness might be to provide more in-depth interaction between the participant and the in-simulation confederate. This might be achieved via an in-depth conversation prior to committing the crime, an activity prior to committing the crime to help set up more of a backstory, and/or more interactions throughout the experience of committing the crime. Performing these types of actions may help participants to develop parasocial bonds to the in-simulation confederate. We plan on investigating this further in future studies.

### The extent of immersion/presence experienced in the virtual reality mock-crimes and associations with intrinsic motivation

Prior studies utilising virtual reality mock-crimes as part of their methodology have reported high levels of realism or presence (e.g.^23,45, 48–52^). Participants in the current study also reported a high level of immersion/presence in both truth and lie conditions for our virtual reality mock-crimes. A high level of immersion/presence is considered important for facilitating participant engagement and motivation to perform a variety of tasks^15,52, 73^.

In our study the hypothesis that higher reported levels of competence, autonomy, and relatedness would be positive associated with higher reported levels of immersion/presence was supported. These positive relationships are consistent with prior studies conducted in the field of education that measured participants’ motivation after completing virtual reality tasks^54,59, 60^. It is unclear from our study what the direction of influence might be. That is, were participants more intrinsically motivated as an influence of higher immersion/presence, or vice versa. Secondly, the correlations between intrinsic motivation and immersion/presence were only moderate (i.e., 0.32–0.52) in magnitude in both truth/lie conditions. Further examination of the influential pathways between the variables of intrinsic motivation and immersion/presence, and other variables related to immersion/presence during virtual reality experiences, are an area for future research.

### Differences in intended effort between the truth and lie conditions

Prior deception detection literature has suggested substantial behavioural and cognitive differences between truth-tellers and liars^19,32, 61–64^. As such, analysis of relationships between variables were separated via the interview condition (i.e., truth/lie). As prior deception detection research has indicated that lying can induce a higher cognitive load^28,31^ we anticipated that deceptive participants would report more intended effort, compared to honest participants. However, the hypothesis that participants in the lie condition would report significantly higher levels of intended effort compared to when they were telling the truth, was not supported. The similarity in reported intended effort seems to indicate that participants desired to perform equally well in the subsequent interview for both conditions. In our study we did not use external motivators (e.g., monetary incentive) nor encouragement to further motivate participants to lie well. Therefore, it may be reasonable to assume that the amount of intended effort was mainly derived from the participants’ own desire to perform well in the interviews, and this was equal across conditions.

## Limitations and future research

In the present study, we recognize that the relationships between intrinsic motivation factors (for the mock-crime) and intended effort (for the interview) were relatively small. However, we also recognize that the design of the mock-crime experiences in this study was quite limited in capacity to facilitate feelings of autonomy, competence and relatedness. There was limited player freedom to perform actions (e.g., they only had a simple task to perform), there was no feedback on performance, and limited interaction with the in-simulation confederate. Therefore, the nature of the mock-crime experiences in our study may not have satisfied participant needs to the extent that we were able to detect stronger associations between self-reported motivation and intended effort. As a result, there was still a large amount of unaccounted variance. As a pilot study examining the role of intrinsic motivation in mock-crimes, we may not have considered other variables that could contribute to predicting effort to perform well in the interview. In the future, we plan on further investigating how more complex virtual mock-crime experiences affect participant motivation and effort.

Another consideration is that the mock-crimes were experienced in virtual reality, but the interview was conducted face-to-face. The switch between the virtual reality mock-crime to an in-person interview could affect the overall role-play immersion. As such, future research is needed to examine if participant self-perceptions of the experience in the interview component might be different across virtual reality versus face-to-face. Lastly, the majority of our participants in this study were not very familiar nor experienced with virtual reality in general. In this study, we did not examine how participants’ familiarity and experience of virtual reality may affect intrinsic motivation, or intended effort as it was outside of our scope of study. However, we encourage other researchers to examine if there could be other potential differences if participants were more experienced or familiar with virtual reality.

## Conclusions

In this study we found evidence that intrinsic motivation experienced in virtual reality mock-crimes affected how much effort participants desired to invest in a subsequent interview phase. While our study was specific to the context of a virtual reality mock-crime, we believe that this finding may be of interest to researchers incorporating mock experiences into their research designs with confederate(s) more broadly; in both virtual reality, and reality. We also found evidence for positive associations between immersion/presence and intrinsic motivation for mock-crime experiences. Based on the findings of this study, more complex virtual reality mock-crime scenarios that have potential to further increase immersion/presence should foster participant intrinsic motivation to enact the mock crime. This should then make the participant feel more inclined to take the overall role-play seriously and put more effort into the subsequent interview phase. We anticipate that developments in technology will make the creation of complex and highly immersive mock-crime experiences easier and more available to researchers in the future.

## Data Availability

The data that support the findings of this study is available 10.6084/m9.figshare.16807444.v8.

## References

[1] Dando CJ, Ormerod TC (2020). Noncoercive human intelligence gathering. J. Exp. Psychol. Gen..

[2] Luke, T. J. & Granhag, P. A. *The Shift-of-Strategy (SoS) Approach: Using Evidence Strategically to Influence Suspects’ Counter-Interrogation Strategies*. 10.31234/osf.io/wncb5 (2020).

[3] Vrij A, Mann S, Leal S, Fisher RP (2020). Combining verbal veracity assessment techniques to distinguish truth tellers from lie tellers. Eur. J. Psychol. Appl. Legal Context.

[4] Alceste F, Jones KA, Kassin SM (2020). Facts only the perpetrator could have known? A study of contamination in mock crime interrogations. Law Hum. Behav..

[5] Dando CJ, Taylor PJ, Sandham AL (2023). Cross cultural verbal cues to deception: Truth and lies in first and second language forensic interview contexts. Front. Psychol..

[6] Dando CJ, Taylor PJ, Menacere T, Ormerod TC, Ball LJ, Sandham AL (2022). Sorting insiders from co-workers: Remote synchronous computer-mediated triage for investigating insider attacks. J. Hum. Factors Ergonom. Soc..

[7] Hoogesteyn K, Meijer E, Vrij A (2020). Examining witness interviewing environments. J. Investig. Psychol. Offend. Profil..

[8] Meenaghan A, Nee C, Van Gelder JL, Vernham Z, Otte M (2020). Expertise, emotion and specialization in the development of persistent burglary. Br. J. Criminol..

[9] van Sintemaartensdijk I, van Prooijen J-W, Nee C, Otte M, van Lange P (2022). Personality and burglary: A virtual reality study. Personal. Individ. Differ..

[10] Romeo T, Otgaar H, Smeets T, Landström S, Jelicic M (2019). The memory-impairing effects of simulated amnesia for a mock crime. Appl. Cogn. Psychol..

[11] Russano MB, Kelly CE, Meissner CA, Bull R, Blandón-Gitlin I (2019). From the ivory tower to the interrogation room. The Routledge International Handbook of Legal and Investigative Psychology.

[12] Nisin Z, Nahari G, Goldsmith M (2022). Lies divorced from context: Evidence for context embedded perception (CEP) as a feasible measure for deception detection. Psychol. Crime Law..

[13] Matsumoto D, Hwang HC (2017). Clusters of nonverbal behaviors differ according to type of question and veracity in investigative interviews in a mock crime context. J. Police Crim. Psychol..

[14] Suchotzki K, Gamer M (2018). Effect of negative motivation on the behavioral and autonomic correlates of deception. Psychophysiology.

[15] Park SY, Lee KH (2021). Burglars’ choice of intrusion routes: A virtual reality experimental study. J. Environ. Psychol..

[16] Hoogesteyn K, Meijer E, Vrij A (2019). The influence of room spaciousness on investigative interviews. Legal Criminol. Psychol..

[17] Chan S, Khader M, Ang J, Chin J, Chai W (2015). To behave like a liar: Nonverbal cues to deception in an Asian sample. J. Police Crim. Psychol..

[18] Geven LM, Ben-Shakhar G, Kindt M, Verschuere B (2019). It’s a match!? Appropriate item selection in the concealed information test. Cogn. Res. Princ. Implic..

[19] Suchotzki K, Verschuere B, Van Bockstaele B, Ben-Shakhar G, Crombez G (2017). Lying takes time: A meta-analysis on reaction time measures of deception. Psychol. Bull..

[20] Kuhns JB, Blevins KR, Bolin RM, Cambareri JF (2017). Drug use and abuse as primary motivators for involvement in burglary: A comparison of self-reported differences among a random sample of male and female burglars. J. Drug Issues.

[21] Sanders AN, Kuhns JB, Blevins KR (2017). Exploring and understanding differences between deliberate and impulsive male and female burglars. Crime Delinq..

[22] Taylor E (2014). Honour among thieves? How morality and rationality influence the decision-making processes of convicted domestic burglars. Criminol. Criminal Just..

[23] Meenaghan A, Nee C, Van Gelder J-L, Otte M, Vernham Z (2018). Getting closer to the action: Using the virtual enactment method to understand burglary. Deviant Behav..

[24] Hudson CA, Vrij A, Akehurst L, Hope L (2019). The devil is in the detail: Deception and consistency over repeated interviews. Psychol. Crime Law.

[25] Jupe LM, Vrij A, Leal S, Nahari G (2019). Fading lies: Applying the verifiability approach after a period of delay. Psychol. Crime Law.

[26] Gálvez-García G, Fernández-Gómez J, Bascour-Sandoval C, Albayay J, González-Quiñones JJ, Moliné A (2020). A trifactorial model of detection of deception using thermography. Psychol. Crime Law.

[27] Verschuere B, Köbis NC, Bereby-Meyer Y, Rand D, Shalvi S (2018). Taxing the brain to uncover lying? Meta-analyzing the effect of imposing cognitive load on the reaction-time costs of lying. J. Appl. Res. Mem. Cogn..

[28] Bird L, Gretton M, Cockerell R, Heathcote A (2019). The cognitive load of narrative lies. Appl. Cogn. Psychol..

[29] DePaulo BM, Lindsay JJ, Malone BE, Muhlenbruck L, Charlton K, Cooper H (2003). Cues to deception. Psychol. Bull..

[30] Maldonado T, Marchak FM, Anderson DM, Hutchison KA (2018). The role of working memory capacity and cognitive load in producing lies for autobiographical information. J. Appl. Res. Mem. Cogn..

[31] Reis M, Pfister R, Foerster A (2022). Cognitive load promotes honesty. Psychol. Res..

[32] Vrij A (2018). Deception and truth detection when analyzing nonverbal and verbal cues. Appl. Cogn. Psychol..

[33] Pérez-Rosas, V., Abouelenien, M., Mihalcea, R. & Burzo, M. Deception detection using real-life trial data. In *Proc. 2015 ACM on International Conference on Multimodal Interaction* 59–66*.*10.1145/2818346.2820758 (Association for Computing Machinery, 2015).

[34] Ryan RM, Deci EL (2000). Intrinsic and extrinsic motivations: Classic definitions and new directions. Contemp. Educ. Psychol..

[35] Ryan RM, Deci EL (2020). Intrinsic and extrinsic motivation from a self-determination theory perspective: Definitions, theory, practices, and future directions. Contemp. Educ. Psychol..

[36] Deci EL, Ryan RM (2000). The “what” and “why” of goal pursuits: Human needs and the self-determination of behavior. Psychol. Inq..

[37] Reis HT, Sheldon KM, Gable SL, Roscoe J, Ryan RM, Reis H (2018). Daily well-being: The role of autonomy, competence, and relatedness. Relationships, Well-Being and Behaviour.

[38] Deci EL, Olafsen AH, Ryan RM (2017). Self-determination theory in work organizations: The state of a science. Annu. Rev. Organ. Psychol. Organ. Behav..

[39] Van den Broeck A, Carpini J, Leroy H, Diefendorff JM, Chmiel N, Fraccaroli F, Sverke M (2017). How much effort will I put into my work? It depends on your type of motivation. An Introduction to Work and Organizational Psychology.

[40] Almagro BJ, Sáenz-López P, Fierro-Suero S, Conde C (2020). Perceived performance, intrinsic motivation and adherence in athletes. Int. J. Environ. Res. Public Health.

[41] Good V, Hughes DE, Kirca AH, McGrath S (2022). A self-determination theory-based meta-analysis on the differential effects of intrinsic and extrinsic motivation on salesperson performance. J. Acad. Market. Sci..

[42] Usán SP, Salavera BC, Teruel P (2019). School motivation, goal orientation and academic performance in secondary education students. Psychol. Res. Behav. Manag..

[43] Howard JL, Bureau J, Guay F, Chong JXY, Ryan RM (2021). Student motivation and associated outcomes: A meta-analysis from self-determination theory. Perspect. Psychol. Sci..

[44] Van den Broeck A, Howard JL, Van Vaerenbergh Y, Leroy H, Gagné M (2021). Beyond intrinsic and extrinsic motivation: A meta-analysis on self-determination theory’s multidimensional conceptualization of work motivation. Organ. Psychol. Rev..

[45] Song I, Kim H, Lee KE, Chang E, Kim HT (2019). Can virtual mock crime replace actual mock crime? An event-related potential study. Korean J. Forensic Psychol..

[46] Coffey AJ, Kamhawi R, Fishwick P, Henderson J (2017). The efficacy of an immersive 3D virtual versus 2D web environment in intercultural sensitivity acquisition. Educ. Technol. Res. Dev..

[47] Gu X, Li S, Yi K, Yang X, Liu H, Wang G (2022). Role-exchange playing: An exploration of role-playing effects for anti-bullying in immersive virtual environments. IEEE Trans. Vis. Comput. Graph..

[48] Mapala T, Warmelink L, Linkenauger SA (2017). Jumping the gun: Faster response latencies to deceptive questions in a realistic scenario. Psychon. Bull. Rev..

[49] Nee C, Gelder J, Otte M, Vernham Z, Meenaghan A (2019). Learning on the job: Studying expertise in residential burglars using virtual environments. Criminology.

[50] Nee C, White M, Woolford K, Pascu T, Barker L, Wainwright L (2014). New methods for examining expertise in burglars in natural and simulated environments: Preliminary findings. Psychol. Crime Law.

[51] Van Gelder J-L, Nee C, Otte M, Demetriou A, van Sintemaartensdijk I, van Prooijen J-W (2016). Virtual burglary. J. Res. Crime Delinq..

[52] Van Gelder J-L, de Vries RE, Demetriou A, van Sintemaartensdijk I, Donker T (2019). The virtual reality scenario method: Moving from imagination to immersion in criminal decision-making research. J. Res. Crime Delinq..

[53] Chen C, Hung H, Yeh H (2021). Virtual reality in problem-based learning contexts: Effects on the problem-solving performance, vocabulary acquisition and motivation of English language learners. J. Comput. Assist. Learn..

[54] Huang W, Roscoe RD, Johnson-Glenberg MC, Craig SD (2020). Motivation, engagement, and performance across multiple virtual reality sessions and levels of immersion. J. Comput. Assist. Learn..

[55] Topîrceanu A (2017). Gamified learning: A role-playing approach to increase student in-class motivation. Procedia Comput. Sci..

[56] Putz L-M, Hofbauer F, Treiblmaier H (2020). Can gamification help to improve education? Findings from a longitudinal study. Comput. Hum. Behav..

[57] Makransky G, Petersen GB (2021). The cognitive affective model of immersive learning (CAMIL): A theoretical research-based model of learning in immersive virtual reality. Educ. Psychol. Rev..

[58] Slater M (2018). Immersion and the illusion of presence in virtual reality. Br. J. Psychol..

[59] Makransky G, Borre-Gude S, Mayer RE (2019). Motivational and cognitive benefits of training in immersive virtual reality based on multiple assessments. J. Comput. Assist. Learn..

[60] Remmer, M., Denami, M. & Marquet, P. Why Pokémon GO is the future of school education. In *Proc. Virtual Reality International Conference—Laval Virtual 2017* 1–5. 10.1145/3110292.3110293 (Association for Computing Machinery, 2017).

[61] Clemens F, Granhag PA, Strömwall LA (2012). Counter-interrogation strategies when anticipating questions on intentions. J. Investig. Psychol. Offend. Profil..

[62] Clemens F, Grolig T (2019). Innocent of the crime under investigation: Suspects’ counter-interrogation strategies and statement-evidence inconsistency in strategic vs non-strategic interviews. Psychol. Crime Law.

[63] Granhag PA, Hartwig M, Giolla EM, Clemens F, Granhag PA, Vrij A, Verschuere B (2014). Suspects’ verbal counter-interrogation strategies. Detecting Deception: Current Challenges and Cognitive Approaches.

[64] Vrij A, Vrij S (2019). Complications travel: A cross-cultural comparison of the proportion of complications as a verbal cue to deceit. J. Investig. Psychol. Offend. Profil..

[65] Branson, I. S., Speelman, C. P. & Rogers, S. More immersive mock-crime experiences in virtual reality facilitates participant effort to be more deceptive in interviews. *figshare. Dataset*. 10.6084/m9.figshare.16807444.v8 (2021).

[66] Geiselman, R. E. The cognitive interview for suspects. *American J. Foren. Psychol.* **30**, 1–16 (2012).

[67] Witmer BG, Jerome CJ, Singer MJ (2005). The factor structure of the presence questionnaire. Presence Teleoper. Virt. Environ..

[68] *Self-Determination Theory*. https://selfdeterminationtheory.org/intrinsic-motivation-inventory/ (n.d.).

[69] Faul, F., Erdfelder, E., Lang, A. G. & Buchner, A. G* Power 3: A flexible statistical power analysis program for the social, behavioral, and biomedical sciences. *Behavior research methods* **39**(2), 175–191. 10.3758/bf03193146 (2007). 10.3758/bf03193146 17695343

[70] Faul, F., Erdfelder, E., Buchner, A. & Lang, A. G. Statistical power analyses using G* Power 3.1: Tests for correlation and regression analyses. *Behavior research methods* **41**(4), 1149–1160. 10.3758/BRM.41.4.1149 (2009). 10.3758/BRM.41.4.1149 19897823

[71] Yee AZH, Sng JRH (2022). Animal crossing and COVID-19: A qualitative study examining how video games satisfy basic psychological needs during the pandemic. Front. Psychol..

[72] Johnson D, Gardner J, Sweetser P (2016). Motivations for videogame play: Predictors of time spent playing. Comput. Hum. Behav..

[73] Cornet LJM, Van Gelder J-L (2020). Virtual reality: A use case for criminal justice practice. Psychol. Crime Law.

